# Commutative algebra neural network reveals genetic origins of diseases

**Published:** 2025-09-30

**Authors:** JunJie Wee, Faisal Suwayyid, Mushal Zia, Hongsong Feng, Yuta Hozumi, Guo-Wei Wei

**Affiliations:** 1 Department of Mathematics, Michigan State University, MI 48824, USA.; 2 Department of Mathematics, King Fahd University of Petroleum and Minerals, Dhahran 31261, KSA.; 3 Department of Mathematics and Statistics, University of North Carolina at Charlotte, Charlotte, NC 28223, USA; 4 Department of Electrical and Computer Engineering, Michigan State University, MI 48824, USA.; 5 Department of Biochemistry and Molecular Biology, Michigan State University, MI 48824, USA.

**Keywords:** mutation, protein solubility, protein stability, genetic disease, commutative algebra

## Abstract

Genetic mutations can disrupt protein structure, stability, and solubility, contributing to a wide range of diseases. Existing predictive models often lack interpretability and fail to integrate physical and chemical interactions critical to molecular mechanisms. Moreover, current approaches treat disease association, stability changes, and solubility alterations as separate tasks, limiting model generalizability. In this study, we introduce a unified framework based on multiscale commutative algebra to capture intrinsic physical and chemical interactions for the first time. Leveraging Persistent Stanley–Reisner Theory, we extract multiscale algebraic invariants to build a Commutative Algebra neural Network (CANet). Integrated with transformer features and auxiliary physical features, we apply CANet to tackle three key domains for the first time: disease-associated mutations, mutation-induced protein stability changes, and solubility changes upon mutations. Across six benchmark tasks, CANet and its gradient boosting tree counterpart, CATree, consistently attain state-of-the-art performance, achieving up to 7.5% improvement in predictive accuracy. Our approach offers multiscale, mechanistic, interpretable,and generalizable models for predicting disease-mutation associations.

## 1 Introduction

Genetic mutations, particularly missense variants, can disrupt protein structure, solubility, and stability, contributing to disease via misfolding and aggregation. These molecular perturbations are implicated in various diseases such as Alzheimer’s disease[1], Parkinson’s disease[2] and amyotrophic lateral sclerosis[3], as well as in cancer[4] and metabolic syndromes[5, 6]. In particular, membrane proteins, which are commonly linked to many diseases such as heart disease, only make up < 0.5% of experimentally resolved structures[7]. Membrane proteins account for 20–30% of human genes and are targets for over half of small-molecule drugs. These proteins play a significant role in cell entry mechanisms, mediating transport, signaling and adhesion, are also disproportionately affected due to their instability outside lipid bilayers[7]. In general, over four million missense variants in the human genome have been identified but only 2% have been clinically classified as pathogenic or benign[8], with the remainder designated as variants of uncertain significance (VUS). Traditional approaches to variant discovery, including whole-genome sequencing (WGS), linkage analysis and genome-wide association studies (GWAS), are time-consuming and resource-intensive[9], requiring extensive data generation, complex filtering and manual curation. Functional validation via cell-based assays or animal models can take weeks to months, and many variants remain unresolved even after substantial effort[10], creating a bottleneck in mechanistic insight and therapeutic development.

Existing predictive methods for identifying disease-associated mutations such as PON-P2[11], SIFT[12], PolyPhen[13], and CADD[14] offer an efficient and potentially reliable alternative to labor-intensive site-directed mutagenesis experiments. However, these models lack the interpretability and may oversimplify structural characterization. Additionally, limited training data with few mutation samples in prior studies like BORODA-TM[15] might have led to model overfitting, with strong validation performance but poor generalization to blind test cases.

Protein stability directly relates protein functions. Computational models for predicting mutation-induced protein stability changes benefit from larger datasets, enabling the application of deep learning architectures such as graph attention networks and multi-task learning algorithms. For example, mutDDG-SSM [16] integrates graph attention networks with gradient boosting trees to achieve high accuracy. Similarly, TNet-MMP-2 [17] employs a multi-task, multi-channel framework to predict disease-associated mutations in globular proteins using protein stability data. Despite these advances, many of these models developed in the past decade, such as STRUM[18], have invoked modern machine learning techniques and leveraged large datasets to uncover hidden relationships between protein stability and protein structure as well as sequence but provide limited interpretability.

Protein solubility is crucial for protein function and human disease. Efforts to predict how mutations affect protein solubility have led to the development of several computational tools as well. Notable examples include CamSol [19], PON-Sol [20], SODA [21], and Solubis [22], which have been comprehensively reviewed in [23]. Building on these, PON-Sol2 [24] introduced an expanded dataset and utilized gradient boosting techniques to enhance sequence-based solubility predictions. Despite these advancements, the overall predictive performance quantified by the normalized Correct Prediction Ratio (CPR) remains suboptimal, indicating a pressing need for more innovative and effective modeling strategies.

Taken together, these observations reveal a critical gap: the absence of a unified framework capable of simultaneously addressing disease-associated mutations, protein stability changes and mutation-induced solubility alterations. Moreover, essential physical interactions, such as hydrogen bonding, van der Waals forces, hydrophobic effects, and electrostatics, have played a significant role in various molecular mechanisms for wild-type functionality and disease-causalities [25, 26, 27]. This also calls for an increasing demand in an eXplainable AI (XAI) framework that improves the interpretability of molecular mechanisms that causes genetic diseases. Bridging these domains could potentially enable the development of more comprehensive, interpretable, mechanistic, and biologically insightful predictive models.

In this work, we introduce multiscale commutative algebra as a novel interpretable algebraic invariant representation to captures mutation-induced intrinsic physical and chemical interactions for the first time. Commutative algebra is deeply rooted in algebraic geometry and number theory [28, 29]and has hardly been applied in data science and machine learning. In this study, we demonstrate for the first time that commutative algebra theory offers a unique tool for revealing intrinsic physical interactions and electrostatic shifts in 3D protein structures. For example, it effectively delineates disruptions to hydrogen bonds and salt bridges, providing an XAI framework for understanding the molecular basis of genetic diseases.

By integrating our multiscale commutative algebra embedding with auxiliary physical features and ESM-2 transformer [30] features, we propose two learning models—Commutative Algebra neural Network (CANet) and Commutative Algebra gradient boosting Tree (CATree), to simultaneously address disease-associated mutations, mutation-induced protein stability changes, and mutation-induced solubility alterations for the first time. Our multiscale commutative algebra embedding applies the Persistent Stanley–Reisner Theory (PSRT), recently introduced by Suwayyid and Wei[31], bridging commutative algebra and multiscale analysis. PSRT instantiates this by analyzing point cloud data through the evolution of square-free monomial ideals in simplicial complexes under filtration, introducing computable algebraic invariants such as persistent facet ideals, f-vectors, h-vectors, and persistent graded Betti numbers. These models simultaneously address three key domains: disease-associated mutation identification, mutation-induced protein stability prediction, and solubility classification. Across six benchmark tasks, our commutative algebra-based models consistently outperform existing approaches, achieving up to 7.5% improvement in predictive performance.

## 2 Results

### 2.1 Overview of CANet workflow

**Figure 1:**
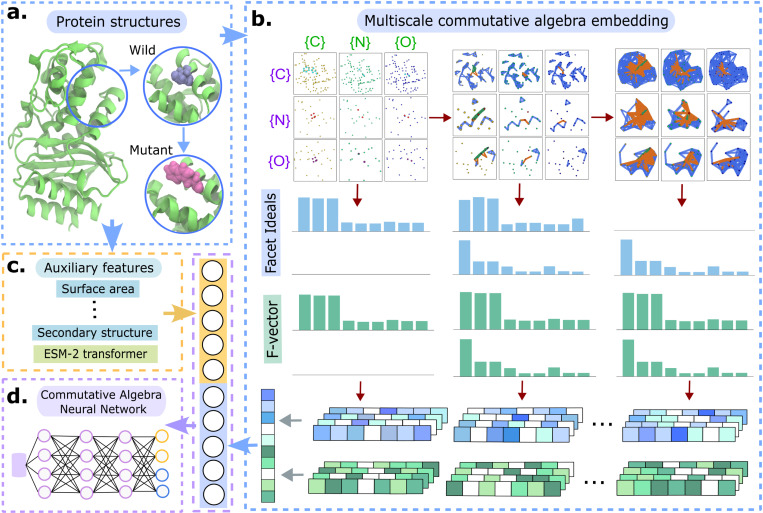
Illustration of commutative algebra neural network (CANet) workflow. **a.** 3D protein structures obtained from the PDB. Mutant proteins are generated from the Jackal software[32]. **b.** Mutational-site and its local neighborhood atom subsets are extracted from both the wild-type and mutant structures to form element specific subcomplexes. Multiscale commutative algebra embedding is performed to generate the persistent facet ideals and persistent f-vector curves. **c.** Auxiliary features such as surface area, secondary structure and ESM-2 transformer-based features are also generated. **d.** Commutative algebra features are concatenated with auxiliary and ESM-2 transformer-based features to form a long feature vector. Features are then fed into the downstream CANet model. The hyperparameters of CANet are optimized. Colors of dotted frames and arrows indicate workflows in different modules: **a.** and **b.** Commutative algebra-based module (blue), **c.** Auxiliary and ESM-2 transformer-based module (orange), **d.** CANet module (purple).

Fig. 1 outlines the workflow of the proposed CANet, which is designed to address three biologically diverse tasks: identifying disease-associated mutations, predicting mutation-induced protein stability changes and protein solubility changes. Given the complexity and heterogeneity of these tasks, designing a unified predictive framework is particularly challenging.

CANet leverages the Persistent Stanley–Reisner Theory (PSRT) [31] to construct multiscale commutative algebra embeddings that encode mutation-induced structural perturbations. In the context of disease-associated mutations, accurate structural characterization is essential for understanding pathogenic mechanisms and guiding therapeutic strategies. PSRT provides a rigorous algebraic framework for capturing subtle yet biologically meaningful changes in protein geometry and electrostatics.

The workflow begins with 3D protein structures from datasets and its mutant structures generated using the Jackal software[32]. From both wild-type and mutant proteins, atom subsets around the mutational site are extracted to form element-specific subcomplexes. These are used to compute persistent facet ideals and f-vector curves under a structural filtration, forming a multiscale commutative algebra embedding. In parallel, auxiliary features—including solvent-accessible surface area, secondary structure annotations, and ESM-2 transformer-based sequence embeddings—are generated. All features are concatenated into a unified representation and passed to the downstream CANet model for training and prediction. A commutative algebra gradient boosting tree (CATree) is also applied as the downstream model, particularly for small datasets.

The interpretable design of CANet enables commutative algebraic features to be traced back to mechanistic insights, such as electrostatic shifts and hydrogen bond disruptions. This biologically grounded eXplainable AI (XAI) framework offers enhanced sensitivity to mutation-induced structural changes, supporting robust and generalizable predictions across diverse protein systems.

### 2.2 Identifying disease-associated mutations

**Figure 2:**
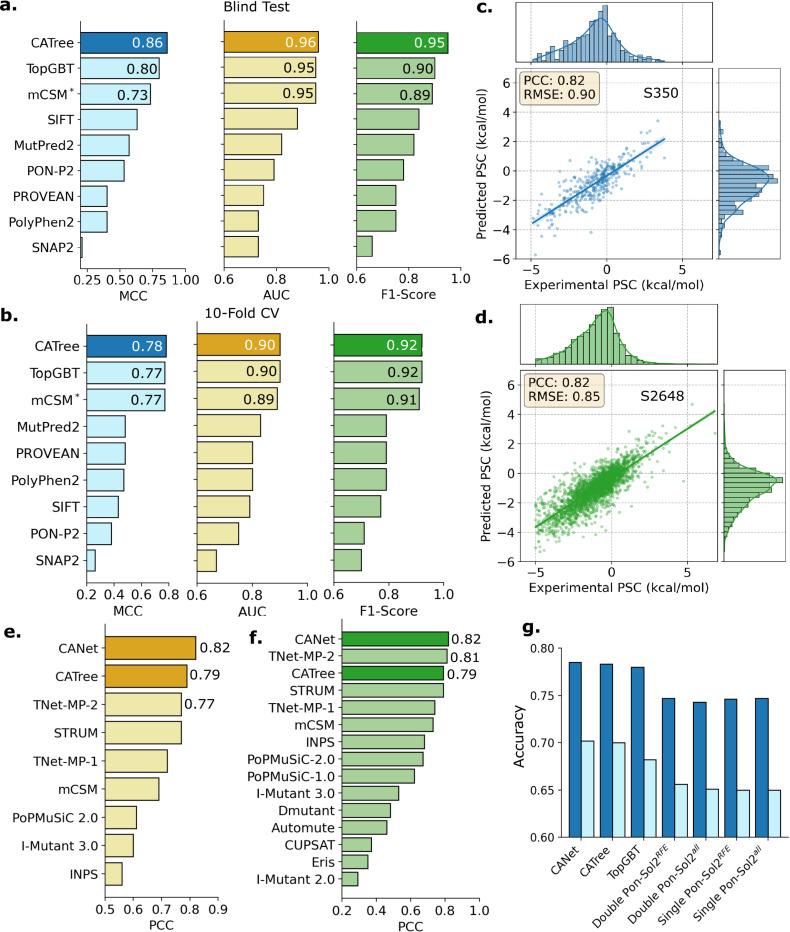
Illustration of commutative algebra model performance in predicting disease-related mutations, protein stability changes and protein solubility changes upon mutation. **a.** Blind test performance of CATree with existing state-of-the-art models [7] in predicting disease-associated mutations. **b.** 10-fold cross-validation performance of CATree in predicting disease-associated mutations. **c.** Comparison of experimental PSC with predicted ones from CANet for S350 dataset. **d.** Comparison of experimental protein stability changes (PSC) with predicted ones from CANet for S2648 dataset. **e.** Performance of CANet and CATree for S2648 dataset compared to existing state-of-the-art models [17, 34, 18]. **f.** Performance of CANet and CATree for S350 dataset compared to existing state-of-the-art models [17, 34, 18]. **g.** Accuracy scores of CANet and CATree for mutation-induced protein solubility change classification compared with existing state-of-the-art models [33, 24]. Dark blue bars represent the accuracy scores and light blue bars are its normalized accuracies.

In this study, we first assess the predictive capability of commutative algebra models in identifying disease-associated mutations. To do this, we conduct a random 10-fold cross-validation alongside an independent blind test using the M546 dataset. This dataset comprises 392 pathogenic and 154 benign mutations derived from 63 transmembrane proteins [15]. Further information about the M546 dataset is provided in Supplementary Information S1. A total of 492 mutations were allocated for training in the cross-validation procedure, while the remaining 54 mutations were reserved for blind testing to rigorously evaluate model effectiveness. Due to the limited size of the dataset and insufficient samples for deep learning, existing models[15, 7] have exhibited signs of overfitting—performing well in cross-validation but failing to generalize in blind testing (see Methods 4.1). To benchmark performance, we also applied TopGBT [33], a persistent homology-based model, to the M546 dataset. As shown in Fig. 2a, CATree outperformed TopGBT in blind test prediction, achieving a Matthews correlation coefficient (MCC) of 0.86, which is 7.5% higher than TopGBT. Furthermore, CATree achieved an area under the ROC curve (AUC) of 0.96 and an F1-score of 0.95, underscoring its robustness and accuracy in predicting mutation effects.

Consistent with the blind test results, CATree also demonstrated strong performance in the 10-fold cross-validation, as shown in Fig. 2b. The model achieved a MCC of 0.78, exceeding that of TopGBT by 1.3%. CATree similarly achieved an AUC of 0.90 and an F1-score of 0.92, demonstrating robustness and predictive accuracy comparable to TopGBT, and outperforming all other existing state-of-the-art models.

### 2.3 Mutation-induced protein stability change prediction

Mutation-induced perturbations in protein stability are a critical molecular mechanism underlying the functional disruption of proteins in many genetic diseases. These changes can alter folding dynamics, interaction interfaces, and overall structural integrity, contributing to pathogenic phenotypes. To further evaluate the predictive performance of our models in this context, we employed the S2648 dataset, which comprises 2,648 mutation samples across 131 protein structures, annotated with mutation-induced changes in protein stability (ΔΔG, kcal/mol)[35]. Model evaluation was conducted through two complementary tasks. First, we performed a 5-fold cross-validation on the full S2648 dataset to assess general performance. Second, we carried out a targeted prediction using the S350 dataset—a curated benchmark subset of S2648—designed specifically for evaluating mutation-induced stability prediction. Predictive accuracy was quantified using the Pearson correlation coefficient (PCC) and root mean squared error (RMSE), providing complementary measures of linear agreement and absolute deviation between predicted and experimental values.

Here, we evaluate both CANet and the Commutative Algebra gradient boosting Tree (CATree). As illustrated in Fig. 2c and 2d, CANet exhibits a strong correlation between predicted and experimentally measured stability changes, highlighting the effectiveness of commutative algebra-based embeddings in capturing mutation-induced structural changes. In the S2648 5-fold cross-validation, CANet surpasses all existing methods, achieving a PCC of 0.82, which represents a 6.49% improvement over TNet-MP-2, a leading topological convolutional neural network model [17] (Fig. 2e). Similarly, CANet achieved a RMSE of 0.85, which is a 9.6% improvement over TNet-MP-2’s result (Supplementary Fig. S4). Furthermore, on the S350 benchmark dataset, CANet maintains its superior performance with a PCC of 0.82, outperforming TNet-MP-2 by 1.23% (Fig. 2f). Notably, CATree also demonstrates competitive performance, outperforming all existing models on the S350 test set except for TNet-MP-2. In contrast to several existing methods, both CANet and CATree are capable of processing all mutation samples in the benchmark datasets, further reinforcing their reliability. For example, I-Mutant 3.0 only evaluated 2636 of 2648 samples in S2648 and 338 of 350 in S350 (see Table S1). These results highlight the robustness and scalability of commutative algebra embeddings, particularly when integrated into CANet, demonstrating accurate predictive performance in mutation-induced protein stability changes.

### 2.4 Mutation-induced protein solubility change classification

Alterations in protein solubility caused by genetic mutations are increasingly recognized as a contributing factor in a range of human diseases, including neurodegenerative disorders, metabolic syndromes, and cancer. Reduced solubility can lead to protein aggregation, misfolding, and impaired cellular function, underscoring the importance of accurately predicting solubility changes upon mutation. To address this, we applied our commutative algebra-based models to the PON-Sol2 dataset[24], which comprises 6,328 mutation samples from 77 distinct proteins. Each sample is annotated with one of three solubility outcomes: decreased, increased, or unchanged. Of these, 3,136 mutations are associated with reduced solubility, 1,026 with increased solubility, and 2,166 show no observable change. The dataset exhibits a pronounced class imbalance (ratio 1:0.69:0.34), with a predominance of mutations leading to decreased solubility (Supplementary Fig. S3c), posing challenges for model training and evaluation.

To assess model performance, we conducted a 10-fold cross-validation and a blind test classification task. Supplementary Fig. S3d shows the distribution of mutation samples across training and test sets in the blind test. In the cross-validation setting, CATree and CANet were benchmarked against TopGBT[33]—a model grounded in persistent homology—and existing PON-Sol2 models[24], which utilize feature selection techniques such as recursive feature elimination (RFE). Given the multi-class nature of the task, normalized accuracy scores were used to provide a comprehensive assessment of predictive performance, as shown in Fig. 2g (light blue bars). CANet and CATree achieved normalized accuracies of 0.702 and 0.700, respectively, representing improvements of up to 7.01% over existing PON-Sol2 models and up to 2.93% over TopGBT.

In the blind test classification task, CANet and CATree maintained consistent performance, achieving normalized accuracies of 0.580 and 0.565, respectively—up to 6.4% higher than PON-Sol2 models and up to 3.2% above TopGBT (Supplementary Fig. S5). To further evaluate model robustness, we employed the generalized squared correlation (GC^2^) metric, which captures nonlinear dependencies between predicted and true labels. Benchmark comparisons based on GC^2^ scores are presented in Supplementary Fig. S6. Similarly, CANet and CATree achieved higher GC^2^ scores, indicating the generalization capability of commutative algebra in class imbalanced tasks.

## 3 Discussion

Having demonstrated the state-of-the-art performance of CANet and CATree across multiple benchmark datasets, we next investigate how specific mutation factors influence predictive accuracy. In particular, we examined the effects of mutation region (e.g., mutations located in interior or surface domains) and mutation type (e.g., hydrophobic-to-polar substitutions) on model performance. These analyses provide insight into the biological contexts in which commutative algebra-based models excel or face limitations. Furthermore, we explore how the algebraic structure underlying our models naturally lends itself to an interpretable, eXplainable AI (XAI) framework by capturing the important electrostatic shifts and physical interactive changes that occur upon mutation. By leveraging the mathematical transparency of commutative algebra, our approach enables the tracing of predictive decisions back to algebraic features, offering a principled pathway toward understanding the molecular basis of variant effects.

### 3.1 Impact of mutation region on predictive performance

**Figure 3:**
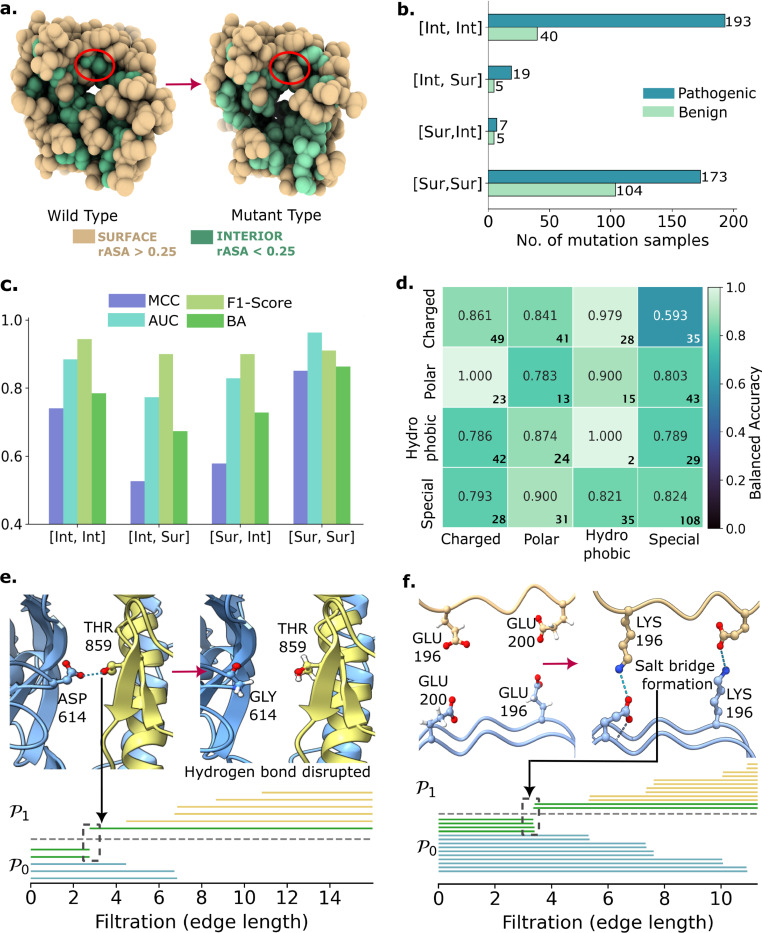
Electrostatic interaction analysis and mutation impact on protein structure and pathogenicity [25]. **a.** Structural shift of the A38E mutation on protein membrane human aquaporin 5 (PDB ID: 3D9S) from surface to interior region, i.e. [Sur, Int]. **b.** The number of pathogenic and benign samples in M546 dataset broken down for each region-region pair. **c.** Balanced accuracy of CATree’s M546 prediction stratified by four mutation region combinations. **d.** Results of CATree’s prediction grouped by amino acid types, showing various impact on balanced accuracy score. Bold numbers indicate sample counts per cell. **e.** Persistent facet ideals reveal hydrogen bonding interactions prior to disruption caused by the D614G mutation in the SARS-CoV-2 spike protein (PDB ID: 6VSB) [36]. Further illustration of the mutation region is depicted in Supplementary Fig. S8. The hydrogen bond are represented by the appearance of the green dimension-1 facet ideal after two green dimension-0 facet ideals stopped persisting at 2.74Å. **f.** Persistent facet ideals illustrate salt bridge formation in the amyloid fibril structure (PDB ID: 7DWV) [37] following mutation E196K linked to genetic prion disease. Dimension-0 facet ideals persist up to 3.35Å and 3.40Å(in green), representing two N–O atom pairs. The emergence of dimension-1 ideals at these distances (in green) marks the formation of a salt bridge, reflecting new electrostatic interactions introduced by lysine.

In this study, we further examine prediction outcomes by categorizing mutation residues based on their mutation regions. Relative accessible surface area (rASA), following the criteria in [38], classifies wild type and mutant residues as either surface-exposed (Sur) or interior (Int) [39] (Fig. 3a). This classification yields four mutation categories: [Int, Int], [Int, Sur], [Sur, Int], and [Sur, Sur], representing combinations of wild-type and mutant residue regions. Fig. 3b presents the sample distribution across these categories for the M546 dataset. This framework, validated in prior studies [33, 40, 41], effectively identifies protein mutation interfaces. The variability in rASA values enables dynamic structural classification, offering insights into mutation adaptability, particularly in disease-associated contexts.

CATree’s predictive performance across the four mutation categories is shown in Fig. 3c. Notably, [Int, Int] and [Sur, Sur] mutations yielded balanced accuracies of 0.78 and 0.86, respectively, outperforming the other two mixed-region categories. A similar performance is observed for the MCC, AUC and F1-Scores, with [Int,Int] and [Sur,Sur] mutations consistently attaining higher scores than the mutations in mixed-region categories. This discrepancy may be attributed to the limited sample size in [Int, Sur] and [Sur, Int] (see Fig. 3b), which can hinder model generalization. An exciting direction for future research involves curating mutation samples with mixed-region categories to improve the model generalization of commutative algebra embeddings and its predictive performance in disease associated mutations.

### 3.2 Impact of mutation types on predictive performance

To assess the influence of biochemical properties on predictive performance, we categorized the 20 canonical amino acids into four mutation types: charged, polar, hydrophobic, and special (see Supplementary Fig. S7 for further details). Fig. 3d presents a heatmap of balanced accuracy values for CATree across all pairwise combinations of these categories. The model maintains consistently high accuracy for most mutation types, with particularly strong performance observed in charged-to-hydrophobic (0.979 with 28 samples), polar-to-charged (1.000 with 23 samples), and hydrophobic-to-polar (0.874 with 31 samples) substitutions. These trends suggest that commutative algebra is highly sensitive to mutations that induce substantial shifts in electrostatic potential or disrupt hydrophobic packing—features often associated with pathogenicity. Conversely, lower accuracy in charged-to-special (0.593 with 35 samples) and polar-to-polar (0.783 with 13 samples) mutations may reflect particularly nuanced and context-dependent physicochemical changes that are harder to resolve.

From Supplementary Fig. S7, 19 of the residues from charged to special are from arginine (R) to tryptophan (W), a substitution known to be structurally disruptive yet highly context-dependent. In transmembrane proteins, R to W mutations can be particularly rare, likely due to the loss of positive charge and the introduction of a bulky, hydrophobic side chain that can perturb membrane insertion, helix packing, or electrostatic anchoring [42]. These residues may possess unique structural roles, including conformational flexibility, disulfide bonding, or helix-breaking tendencies, which are often context-dependent and not easily captured by model generalization. Similarly, polar-to-polar mutations often preserve hydrogen bonding potential and side-chain polarity, resulting in minimal changes to the protein’s electrostatic landscape. These nuanced alterations may not produce strong geometric or energetic perturbations, making them more difficult for commutative algebra models to distinguish as pathogenic or benign. The accompanying few sample counts further contextualize these results, highlighting the importance of residue-specific representation in understanding mutation effects. Overall, this analysis underscores the utility of commutative algebraic models in capturing the geometric and biochemical disruptions that underlie disease-associated mutations.

### 3.3 Commutative algebra enabled eXplainable AI

Understanding the origin of genetic diseases requires a rational learning framework capable of interpreting the biological and structural mechanisms underlying pathogenic mutations. In this study, we proposed CANet as an eXplainable AI (XAI) model that leverages commutative algebra to represent protein structures and infer mutation-induced perturbations. CANet achieves interpretability through algebraic embeddings based on persistent Stanley–Reisner rings, which encode multiscale facet ideals and f-vector features derived from protein structural filtrations. These representations naturally bridge a significant gap between computational biophysics and XAI, providing mechanistic insights into how specific mutations can lead to diseases, alter protein stability and solubility.

#### Protein properties captured by commutative algebra.

To demonstrate the interpretability of CANet, we traced its commutative algebraic representations—specifically, persistent facet ideals—back to biologically meaningful protein functionalities that govern electrostatic interactions and conformational stability. This analysis reveals that algebraic invariants serve as eXplainable AI (XAI) frameworks capable of elucidating the critical role of electrostatics in disease-associated mutations. In the SARS-CoV-2 spike protein, for instance, CANet identifies hydrogen bonds (average distances ∼3.0Å) between residues T859 and D614 (Fig. 3e). The D614G mutation, a defining feature of early viral evolution, disrupts this interaction, contributing to enhanced infectivity during the initial wave of the COVID-19 pandemic [43]. In Fig. 3e, two dimension-0 facet ideals (green bars) persist up to 2.74Å, corresponding to oxygen atoms from ASP614 and THR859. At this threshold, a dimension-1 ideal emerges, indicating hydrogen bond formation—an interaction lost upon mutation, thereby altering viral fitness.

A similar pattern is observed in transmembrane protein human aquaporin AQP5 (Supplementary Fig. S9), where a charged mutation (A38E) disrupts hydrogen bonding between A38 and Y178. This is consistent with prior studies, which have shown that A38E perturbs the local electrostatic environment, affecting loop conformation and ultimately altering the protein’s tertiary and quaternary structure [44].

Further, Fig. 3f illustrates a salt bridge (∼3.40Å) formed in the amyloid fibril structure following the E196K mutation, which is associated with genetic prion disease. The substitution introduces a positively charged lysine that engages in electrostatic and hydrogen bond interactions with glutamic acid, stabilizing the fibril through salt bridge formation. As an extension, algebra invariants can be potentially applied to extract interpretable co-evolutionary structural changes, which greatly influence the protein’s mutational pathways[45], developing distinct persistent facet barcodes for each scenario (Supplementary Fig. S10).

#### Structural motifs captured by commutative algebra.

**Figure 4:**
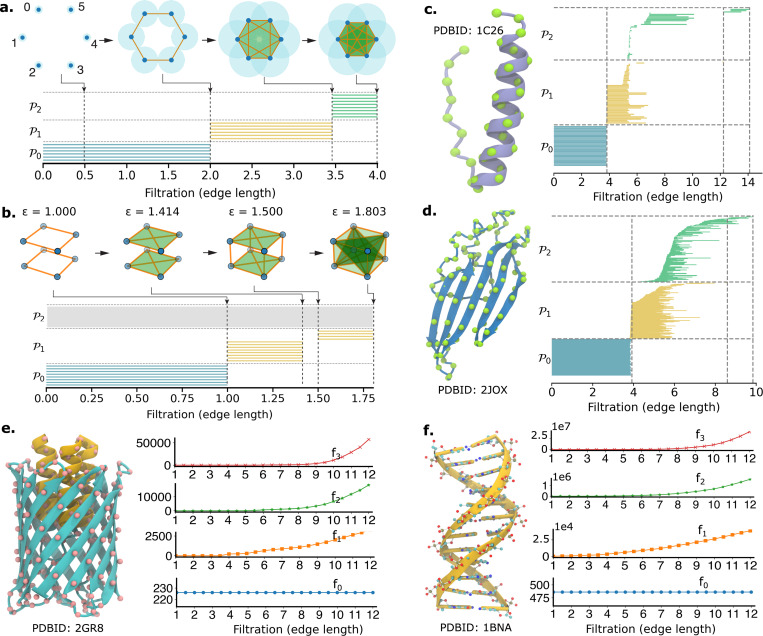
Illustrations of multiscale commutative algebra analysis on point-cloud data using a Rips complex-based filtration process: a. Facet persistence barcode for 6 points. b. Facet persistence barcode for a cuboid with dimensions 1 × 1 × 1.5. c. Facet persistence barcode for the $C_{\alpha }$ atoms of protein 1C26 with alpha-helix structures. d. Facet persistence barcode for the $C_{\alpha }$ atoms of protein 2JOX with beta-sheet structures. e. f-vector curves for the $C_{\alpha }$ atoms of protein 2GR8. f. f-vector curves for the atoms in DNA structure 1BNA.

To illustrate, we first perform multiscale commutative algebra analysis on point-cloud data using a Rips complex-based filtration process, as depicted in Figure 4. The upper panel of Figure 4a presents a case study of a six-point configuration, while the lower panel displays the corresponding persistent facet ideals for dimensions 0, 1, and 2. In dimension 0, six facet bars persist up to a filtration parameter (edge length) of 2, corresponding to the six disconnected points that subsequently merge into six edges (1-simplices) at this value. At a filtration value of 3.5, these edges form the boundaries of triangles (2-simplices), resulting in the disappearance of the six yellow bars in dimension 1. As the filtration parameter increases from 3.5 to 4, additional higher-dimensional simplices, such as 4-simplices, emerge. As a result, the triangles observed in the third filtration stage become the faces of these higher-order simplices, leading to the persistence of the corresponding facet barcodes from filtration values 3.5 to 4. In the drawing barcode, we exclude those with persistence length equal to 0.

Figure 4b shows the filtration process and the corresponding facet persistence bars for a cuboid with dimensions 1×1×1.5. Similar to the previous example, six facet bars (shown in blue) for dimension 0 persist up to a filtration parameter (edge length) of 1, as the eight initially independent points become connected by edges with the minimal edge length of 1. There are two groups of yellow facet bars for dimension 1. The first group, consisting of 8 bars, represents features that appear in the initial stage of the filtration and disappear in the second stage. The second group, with 4 yellow bars, corresponds to the four vertical edges that emerge in the third stage of the filtration process and vanish in the fourth stage as they merges into triangular faces. No bars of dimension two are observed due to the simultaneous formation of those triangles and their enclosing tetrahedra.

Next, we illustrate the interpretability of CANet using fundamental structural motifs. Figure 4c displays the facet persistence bars across three dimensions for the $C_{\alpha }$ atoms in an alpha-helix protein (PDB ID: 1C26). The blue bars on the right panel indicate that these $C_{\alpha }$ atoms connect within a filtration value of 4Å, consistent with typical physical distances between adjacent $C_{\alpha }$ atoms. The yellow bars (dimension 1) emerge around 4Å, corresponding to edge connections between neighboring $C_{\alpha }$ atoms. Due to the close spatial proximity of any three consecutive $C_{\alpha }$ atoms along the alpha-helix, triangular structures form rapidly, resulting in short-lived dimension-1 features. Most of these bars disappear around 5Å, reflecting the compact geometry of the helix, while a few persist until 6.5Å, primarily involving terminal regions where longer connections are required. The green bars (dimension 2) correspond to the persistence of triangular faces. In the alpha-helix region, these triangles quickly evolve into tetrahedra, resulting in short persistence intervals, typically between 5Å and 6Å. The remaining green bars, with death values extending up to 14Å, are associated with tetrahedra formed between terminal atoms and those in the helix. The broader range of births and deaths for dimension-2 features, especially between 5Å and 14Å, reflects the geometric variability in connecting alpha-helix and terminal regions.

Figure 4d presents the facet persistence analysis for a beta-sheet protein structure (PDB ID: 2JOX) containing 106 $C_{\alpha }$ atoms. Similar to the alpha-helix case, most dimension-0 bars die around 4Å due to the short distances between neighboring $C_{\alpha }$ atoms. The larger number of atoms results in more edges and triangles, leading to a higher number of persistence bars overall. As before, most yellow bars (dimension 1) appear between 4Å and 6Å. However, the green bars (dimension 2) tend to persist longer than in the alpha-helix case, as $C_{\alpha }$ atoms in beta-sheets are less likely to form tetrahedra as readily as those in helical structures.

The membrane protein in Figure 4e contains 226 $C_{\alpha }$ atoms. The large number of atoms makes the persistent facet barcode analysis computationally impractical, due to the excessive number of edges, triangles, and tetrahedra formed. As an alternative, the f-vector is well-suited for structural data analysis, as it counts the number of simplices of each dimension at different filtration stages. The results show tens of thousands of tetrahedra forming as the filtration radius increases, while the dimension-0 f-vector curve remains constant since the number of atoms does not change. A similar situation occurs for the DNA structure (PDB ID: 1BNA) in Figure 4f, which contains 486 atoms. Here, the number of triangles and tetrahedra can reach magnitudes of 10^6^–10^7^ at a filtration radius of 12Å, making facet barcode analysis infeasible. In both cases, f-vector analysis provides a scalable alternative, though it comes at the cost of losing detailed persistence information.

As a result, CANet introduces a biologically grounded, algebraically driven XAI framework that enables interpretable modeling of mutation-induced structural and electrostatic perturbations in proteins. Compared to persistent homology—a well-established method in topological data analysis (TDA) that captures global features such as loops and cavities—commutative algebra provides enhanced sensitivity to local structural changes and electrostatic perturbations. While persistent homology identifies coarse-grained topological invariants, it often misses subtle but biologically critical interactions. In contrast, commutative algebraic descriptors, including facet ideals and f-vector features, encode multiscale geometric and chemical information. These capabilities enable CANet to detect fine-grained alterations in protein structure and function, highlighting commutative algebra’s strength as a rigorous and interpretable XAI framework for uncovering the molecular basis of genetic diseases.

## 4 Methods

In this section, we outline the data collection, multiscale commutative algebra embedding framework and its feature generation for wild-type and mutant protein structures. Computational settings for CANet and CATree is also provided. Details on auxiliary features, ESM-2 descriptors and performance metrics can be found in the Supplementary Information.

### 4.1 Data Collection

To rigorously assess the performance of CANet and CATree, we curated a diverse set of benchmark datasets spanning three core tasks related to mutation-induced effects on protein structure and function: Disease-associated mutations, protein stability changes and protein solubility changes.

#### M546 dataset

The M546 dataset originated from the BORODA-TM[15] study, which focuses on disease-associated mutations in transmembrane proteins with known 3D structures. It comprises of 392 pathogenic and 154 benign point mutations across 64 transmembrane proteins. The breakdown of pathogenic and benign point mutations for M546 are summarized in Supplementary Fig. S3a and b. Notably, 492 mutations are used for the 10-fold cross-validation while the remaining 54 samples are used in a blind test prediction. These mutations were curated from UniProtKB/SwissProt and mapped to structural data from the Human Transmembrane Proteome (HTP) database. The dataset emphasizes mutations located within transmembrane helices, which are particularly relevant for understanding membrane protein dysfunction in disease.

It is important to note that a recent study from mCSM[7] revealed a marked discrepancy between cross-validation (MCC = 0.87, F1-Score = 0.96) and blind test performance from BORODA-TM (MCC = 0.46, F1-Score = 0.78), suggesting potential overfitting and underscoring the need for robust generalization. In addition, mCSM[7] only successfully processed 539 samples as 7 samples from PDB ID: 4ZWJ[46] contains missing residues in the mutation site.

#### S2648 and S350 dataset

The S2648 dataset[35] contains 2,648 single-point mutations across 132 proteins, annotated with experimentally measured changes in protein stability. It serves as a comprehensive benchmark for regression-based prediction of mutation-induced stability changes. The S350 dataset is a curated subset of S2648, consisting of 350 mutations in 67 proteins, and is commonly used for blind testing and comparative evaluation of predictive models.

#### PON-Sol2 dataset

The PON-Sol2 dataset[24] includes 6,328 mutations across 77 proteins, annotated with protein solubility changes: increased, decreased, or neutral (unchanged). The breakdown of mutation samples by their solubility changes for M546 are summarized in Supplementary Fig. S3c and d. It was developed to support machine learning-based prediction of mutation-induced solubility changes. The dataset addresses class imbalance and includes both experimentally validated and literature-curated variants. It is widely used for multi-class classification tasks in protein solubility prediction.

### 4.2 Multiscale commutative algebra embedding

#### 4.2.1 Element-specific and Site-specific Atom sets

In this work, we construct multiscale commutative algebra embeddings using element- and site-specific atom sets. This approach simplifies protein geometry while capturing key physical interactions through algebraic invariants.

Atoms in a 3D protein structure are partitioned into site-specific atom sets: mutation-site atoms ($A_{m}$) and mutation neighborhood atoms within a cutoff radius r, denoted as $A_{mn}(r)$. We further classify atoms into element-specific subsets, i.e. $A_{\varepsilon }$, where ε∈{C,N,O}. This yields nine distinct pairwise combinations between mutation site and mutation neighborhood atoms. These combinations reflect distinct interaction types—for example, $A_{C}\cap A_{m}$ and $A_{C}\cap A_{mn}(r)$ encode hydrophobic C–C interactions, while $A_{N}\cap A_{m}$ and $A_{O}\cap A_{mn}(r)$ capture hydrophilic N–O interactions, including hydrogen bonds and salt bridges.

To accurately capture interactions between mutation sites and their surrounding atomic environments, we also have to modify the standard Euclidean distance so that it exclude interactions between both atoms found in the $A_{m}$ or both in the $A_{mn}(r)$. For example, for interactions between atoms $a_{i}$ and $a_{j}$, we connect the atoms using the following modified Euclidean distance ${\text{D}}_{\mathrm{mod}}$:

(1) $${\text{D}}_{\text{mod}}\left(a_{i},a_{j}\right)=\left\{\begin{array}{ll} \infty , & \text{either}\;a_{i},a_{j}\in A_{m}\;\text{or}\;a_{i},a_{j}\in A_{mn}(r)\\ \mathrm{DE}\left(a_{i},a_{j}\right), & \text{otherwise}. \end{array}\right.$$

Here, DE(⋅,⋅) refers to the Euclidean distance between two atoms.

#### 4.2.2 Persistent Stanley–Reisner Structures over a Filtration

Using a single simplicial complex alone is insufficient to extract all the algebraic invariants into structural features. By combining commutative algebra and multiscale analysis, we track the variations of facet ideals and f-vectors by adjusting a filtration parameter such as radii/diameter for VR complex [31]. For an oriented simplicial complex Δ, a filtration creates a nested sequence of simplicial complexes ${\left({\text{Δ}}^{t}\right)}_{t=0}^{m}$ of Δ,

$$\emptyset ={\text{Δ}}^{0}\subseteq {\text{Δ}}^{1}\subseteq \cdots \subseteq {\text{Δ}}^{m}=\text{Δ}.$$

As the value of the filtration parameter increases, this generates a sequence of simplicial complexes. Based on this nested sequence of simplicial complexes, we can produce a descending filtration of Stanley-Reisner structures

$$I^{0}⊇I^{1}⊇I^{2}⊇I^{3}⊇\cdots ⊇I^{m}\;\text{where}\;I^{t}:=I\left({\text{Δ}}^{t}\right)\;\text{for}\;1\leq t\leq m.$$

Denote $F\left({\text{Δ}}^{t}\right)$ as the set of all facets in the filtered subcomplex ${\text{Δ}}^{t}$. By adjusting the filtration parameter and canonically decompositioning these Stanley-Reisner structures, each Stanley-Reisner structure $I^{t}$ yields an intersection of prime monomial ideals $P_{\sigma }$ associated to the facets of ${\text{Δ}}^{t}$. In other words, $P_{\sigma }$ consists of the facet ideals at filtration value t. At each filtration value t, the collection

$$P_{i}^{t}:=\left\{P_{\sigma }|\sigma \in F\left({\text{Δ}}^{t}\right)\;\text{and}\;\dim(\sigma )=i\right\},$$

contains all the facet ideals at filtration value t of face dimension i. Thereafter, we can count the number of i-dimensional facet ideals at each filtration level t.

Similarly, we also can count the number of i-dimensional faces in ${\text{Δ}}^{t}$. This generates an f-*vector* of ${\text{Δ}}^{t}$ which can be written as

$$f\left({\text{Δ}}^{t}\right)=\left(f_{-1}^{t},f_{0}^{t},f_{1}^{t},\ldots ,f_{d-1}^{t}\right),$$

where $f_{i}^{t}$ is the number of i-dimensional faces in ${\text{Δ}}^{t}$, and $f_{-1}^{t}=1$ by convention.

Ultimately, these commutative algebraic descriptors help CANet and CATree track the changes of algebraic invariants in 3D protein structures, capturing both the facet ideals and f-vectors throughout its filtration process. Further details of persistent Stanley-Reisner theory is provided in Supplementary Information S1.

### 4.3 Commutative algebra feature generation

Using the element-specific, site-specific atom subsets, we generate commutative algebraic features via Vietoris–Rips (VR) and Alpha complexes, from both the wild-type and mutant protein structures. The Vietoris–Rips complex forms simplices from atoms with pairwise distances below a threshold, while the Alpha complex, derived from Delaunay triangulation, restricts simplices to those enclosed within a specified radius, subdividing the convex hull of a point set into triangles. The modified Euclidean distance D_mod_ is used to construct the VR complexes while the standard Euclidean distance is used for the Alpha complexes. In this study, a cutoff distance of 16Å from the mutation site is used to collect mutation neighborhood atoms. The filtration range is set from 1Å to 12Å for each case, with a step size of 0.4Å for the filtration steps. For each filtration, the 0 and 1-dimensional facet ideals and f-vectors are calculated at each step size and concatenated to form our multiscale commutative algebra embedding. To illustrate, Supplementary Fig. S1 and S2 depicts the facet ideals and f-vector curves from protein ID: 213133708 of the PON-Sol2 dataset. Fig. S1 uses atom sets $A_{C}\cap A_{m}$ and $A_{C}\cap A_{mn}(r)$ to generate VR complexes with D_mod_-based filtration, revealing the hydrophobic C-C interactions. Similarly, Fig. S2 uses atom sets $A_{N}\cap A_{m}$ and $A_{O}\cap A_{mn}(r)$ to generate VR complexes with D_mod_-based filtration, revealing the N-O hydrophillic and/or hydrogen bond interactions. In our commutative algebra embeddings, we also consider the difference between the wild-type and mutant in both the facet ideals and f-vector curves.

### 4.4 CANet architecture and CATree model hyperparameters

The deep neural network model in CANet’s architecture consists of six hidden layers with 15,000 neurons in each layer and generates either regression output or classification label depending on the prediction task. For the protein stability change prediction tasks, the batch size is 32. The learning rate is set to 0.001 and 200 epochs are used for the training step. For the protein solubility change classification tasks, the batch size is 50. The learning rate is also set to 0.001 and 200 epochs are used for the training procedure.

In this study, we also used a Commutative Algebra gradient boosting Tree (CATree) model to handle small datasets like M546, which contains limited mutational samples for training in deep neural networks. The gradient boosting trees (GBT) are implemented using the Python scikit-learn package (v1.3.2) for implementation [47]. GBTs are well-regarded for its robustness against overfitting, relative insensitivity to hyperparameter settings, and ease of implementation. The algorithm creates multiple weak learners or individual trees by bootstrapping training samples and integrates their outputs to make predictions. Although weak learners are prone to making poor predictions, the ensemble approach can reduce overall errors by combining the predictions of all the weaker learners. We input resulting commutative algebraic descriptors and ESM-2 transformer features into the CATree algorithm to build regression models, respectively. The CATree hyperparameters used for modeling are listed in Table 1.

## Figures and Tables

**Table 1:** CATree model hyperparameters.

No. of estimators	Max depth	Min. sample split	Learning rate
20000	7	3	0.05

Max features	Subsample size	Repetition	
Square root	0.4	10 times	

## Data Availability

All datasets analyzed in this study are publicly available from the sources cited in the manuscript. 3D protein structures are available at https://github.com/ExpectozJJ/CAN.

## Code Availability

The implementation of the proposed CANet and CATree framework is available at https://github.com/ExpectozJJ/CAN including the source code for the methods used for comparison in this study.
